# Non-invasive Estimation of Foliar Nitrogen Concentration Using Spectral Characteristics of Menthol Mint (*Mentha arvensis* L.)

**DOI:** 10.3389/fpls.2022.680282

**Published:** 2022-05-09

**Authors:** Praveen Pandey, Swati Singh, Mohammad Saleem Khan, Manoj Semwal

**Affiliations:** ^1^Information and Communication Technology Department, CSIR–Central Institute of Medicinal and Aromatic Plants, Lucknow, India; ^2^Academy of Scientific and Innovative Research (AcSIR), Ghaziabad, India

**Keywords:** *Mentha arvensis* L., foliar nitrogen, spectral reflectance, vegetation index, partial least squares regression

## Abstract

Menthol mint (*Mentha arvensis* L., Family: Lamiaceae), popularly known as corn mint or Japanese mint, is an important industrial crop that is widely grown for its valued essential oil. Nitrogen (N) is an important macro-nutrient and an essential factor for optimizing the yield and quality of crops. Hence, rapid and accurate estimation of the N content is crucial for nutrient diagnosis in plants and to make precise N fertilizer recommendations. Generally, N concentration is estimated by destructive sampling methods; however, an indirect assessment may be possible based on spectral characteristics. This study aimed to compare the foliar N concentration based on non-destructive (reflectance) and destructive (laboratory analyses) methods in menthol mint. Foliar N concentration was measured through the Kjeldahl method and reflectance by Miniature Leaf Spectrometer C-710 (CID Bio-Science). Using reflectance data, several vegetation indices (VIs), that is, normalized difference red edge (NDRE), red edge normalized difference vegetation index (reNDVI), simple ratio (SR), green–red vegetation index (GRVI), canopy chlorophyll content index (CCCI), photochemical reflectance index (PRI), green chlorophyll index (CI_*Green*_), red edge chlorophyll index (CI_*Red Edge*_), canopy chlorophyll index (CCI), normalized pigment chlorophyll ratio index (NPCI), and structure insensitive pigment index (SIPI), were developed to determine the foliar N concentration. The highest correlation (r) between VIs and foliar N concentrations was achieved by NDRE (0.89), followed by reNDVI (0.84), SR (0.83), GRVI (0.78), and CCCI (0.76). Among the VIs, the NDRE index has been found to be the most accurate index that can precisely predict the foliar N concentration (*R*^2^ = 0.79, RMSE = 0.18). In summary, the N deficiencies faced by the crop during its growth period can be detected effectively by calculating NDRE and reNDVI, which can be used as indicators for recommending precise management strategies for the application of nitrogenous fertilizers.

## Introduction

Menthol mint (*Mentha arvensis* L., Family: Lamiaceae), popularly known as corn mint or Japanese mint, is a short-term (90–110 days) cash crop that is widely grown for its valued essential oil and provides livelihood support to millions of smallholder farmers. India is the principal producer and supplier of mint oil in the world (about 80% global share), followed by China, Brazil, and the United States (Khan et al., 2020). The high demand for menthol mint oil in the global market in the last few years has drastically increased its cultivation capacity, with around 2,50,000 hectares of land being cultivated by nearly 5,00,000 farming families in India (Khan et al., 2020). The economic security of this crop relies on farmers’ prosperity, which can be accomplished by enhancing the productivity of their farms with minimal inputs. Among the various aspects of farm inputs, fertilizers play a vital role in improving the productivity of food and commercial crops. The spatial and temporal variability in soil and nutrient requirements is not uniform even within the same field, which can be attributed to inherent soil properties, peculiar nutrient supply, and versatile crop management practices (Dobermann et al., 1996). Therefore, site-specific nutrient assessment is crucial for sustainable crop production.

Nitrogen (N) is one of the most important macro-nutrients essential for plant growth, development, and quality of the crop (Clevers and Gitelson, 2013; Biswas and Ma, 2016). Plant growth and development is not static but a dynamic process that requires persistent N throughput (Kattge, 2002). N regulates a range of cellular functions, such as growth, absorption, transportation, excretion, etc. Moreover, it is also a major constituent of amino acids that are the building blocks of proteins. In addition, it indirectly helps in the process of photosynthesis *via* chlorophyll production (Li et al., 2014). On one hand, insufficient N supply to the plants can cause damage to the photosynthesis process, thus resulting in reduced biomass and yield. On the other hand, its excessive use can degrade soil and environmental quality, and hence an appropriate amount of N must be supplied. However, farmers apply excess amounts of nitrogenous fertilizers to their fields to obtain higher yields without knowing its harmful effects on soil and environmental health (Ju et al., 2006). Therefore, continuous monitoring of this key plant characteristic and precise N fertilization (adequate rate and time) are crucial for increasing agricultural productivity while preserving environmental sustainability.

The traditional method for N measurement is tedious and time consuming, and is typically performed by destructive sampling and laboratory analyses, making it difficult to fulfill the challenges of precise crop management in large-scale agricultural fields. In recent years, remote sensing technologies have been established as effective methods for the non-destructive detection of N concentration in several crops, such as rice (Tian et al., 2011; Du et al., 2016; Yang et al., 2016; Sun et al., 2017; Zhou et al., 2018), wheat (Hansen and Schjoerring, 2003; Zhu et al., 2007; Feng et al., 2014; Li et al., 2014; Yao et al., 2015), oilseed rape (Li et al., 2018), cotton (Tarpley et al., 2000), soybean (Song and Wang, 2016), and eucalyptus (Oliveira et al., 2017). It provides comprehensive dimensions of vegetation indices (VIs) consistent with the optical function of biochemical foundations, which have a theoretical advantage over traditional measurements for the detection of N in modern agriculture. Over the years, empirical regression algorithms have been predominantly utilized for the N assessment based on the vegetation spectral signatures in the agronomic context. These methods are the most useful assessment systems that are based on major biophysical and biochemical vegetation characteristics. Regression models evaluate different narrowband VIs using wavelengths primarily in the NIR, red edge, and SWIR regions (Chen et al., 2010; Jay et al., 2017). Studies have shown that N estimation can be related to spectral bands associated with chlorophyll absorption due to the similarity in the region of absorption, as N is predominantly localized in the building blocks of chlorophyll (Baret et al., 2007; Schlemmer et al., 2013). Subsequently, differences in chlorophyll intensities result in substantial spectral variations, particularly in the red-edge region, which is a critical component of the vegetation spectrum (Main et al., 2011). Several reports have shown better retrievals of foliar N when protein-linked absorption bands in the NIR and SWIR regions were brought together with different narrowband VIs (Serrano et al., 2002; Herrmann et al., 2010).

Although N assessments in different crops have been widely reported, only a few efforts have been made in the menthol mint crops (Singh et al., 2019). Therefore, more oriented research is required to develop a precise and robust method intended to estimate plant N status with high consistency and practicability and to determine the N requirement of crops using different sensors. Thus, this study aimed to explore different VIs and assess their relationship with foliar N content in menthol mint crops for precise N fertilizer management.

## Materials and Methods

### Site Description

**FIGURE 1:**
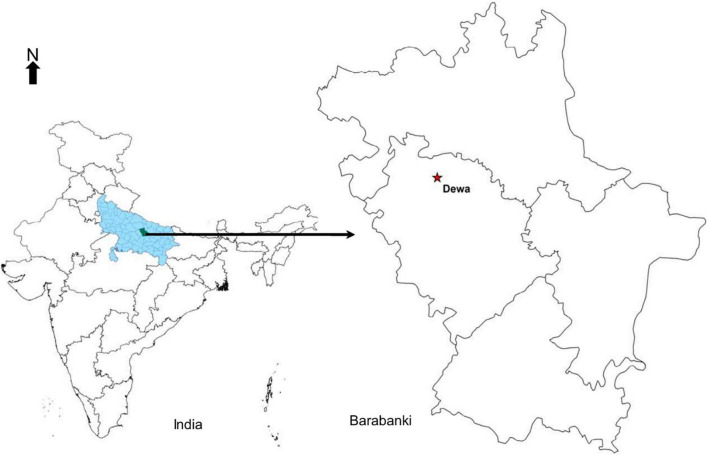
Location of the experimental site.

The field experimentation site (Figure 1) is located in Barabanki, Uttar Pradesh, India (27°03′N, 81°17′E). It is a part of the Indo-Gangetic Plains with a sub-humid climate and sandy loam soil. The study area, Barabanki, has emerged as a major hub for the production of menthol mint in the world, alone contributing to about 60% of the global share. Apart from menthol mint, other major crops cultivated in the district are paddy, wheat, maize, potato, mustard, pigeon pea, okra, chilies, etc. (Khan et al., 2020). In the present investigation, *Mentha arvensis* cv. Kosi, developed by CSIR-Central Institute of Medicinal and Aromatic Plants, was used to determine the correlation between different VIs and the foliar N concentration based on non-destructive (reflectance) and destructive (laboratory analyses) methods.

### Foliar Nitrogen Estimation

For field destructive sampling, to represent the amount of canopy N, the established leaves which are most recent and expanded first below the growing point were collected for the study. In each geo-referenced sampling point, we selected 15–20 leaves for total N analysis in this study. Leaf samples were oven-dried (70°C) and powdered, and were subjected to wet digestion with HNO_3_ + H_2_O_2_ by the Kjeldahl method to assess the N content (Lang, 1958).

### Measurements of Leaf Spectral Reflectance Indices

The spectral reflectance in the range of 400–1,000 nm was measured using a C-710 Miniature Leaf Spectrometer (CID Bio-Science) and analyzed using SpectraSnap! software. VIs were extensively utilized to differentiate plant nutrient concentration, and the selection of different VIs used in this study was based on their capacity to retrieve chlorophyll and N content according to the methods described in the literature. The following VIs were measured through reflectance spectra: normalized difference red edge (NDRE), red edge normalized difference vegetation index (reNDVI), simple ratio (SR), green–red vegetation index (GRVI), canopy chlorophyll content index (CCCI), photochemical reflectance index (PRI), green chlorophyll index (CI_*Green*_), red edge chlorophyll index(CI_*Red Edge*_), canopy chlorophyll index (CCI), normalized pigment chlorophyll ratio index (NPCI), and structure insensitive pigment index (SIPI) (Table 1).

**TABLE 1:** Different vegetation indices (VIs) used in this study.

Vegetation indexes	Short	Formula	References
Normalized difference red edge	NDRE	(ρ800−ρ720)/(ρ800 + ρ720)	Rouse et al., 1973
Red edge normalized difference vegetation index	reNDVI	(ρ750−ρ705)/(ρ750 + ρ705)	Gitelson and Merzlyak, 1994
Simple ratio	SR	(ρ800/ρ670)	Birth and McVey, 1968
Green-red vegetation index	GRVI	(ρ800/ρ560)	Tucker, 1979
Canopy chlorophyll content index	CCCI	[(ρ840−ρ717)/(ρ840 + ρ717)]/[(ρ840−ρ668)/(ρ840 + ρ668)]	Fitzgerald et al., 2006
Photochemical reflectance index	PRI	(ρ531−ρ570)/(ρ531 + ρ570)	Gamon et al., 1997
Green chlorophyll index	CI_*Green*_	(ρ730/ρ530)-1	Gitelson et al., 2003, 2005
Red edge chlorophyll index	CI_*Red Edge*_	(ρ850/ρ730)-1	Gitelson et al., 2003, 2005
Canopy chlorophyll index	CCI	(ρ720/ρ700)	Sims et al., 2006
Normalized pigment chlorophyll ratio index	NPCI	(ρ680−ρ430)/(ρ680 + ρ430)	Peñuelas et al., 1994
Structure insensitive pigment index	SIPI	(ρ800−ρ445)/(ρ800–ρ680)	Peñuelas et al., 1995

### Statistical Analysis and Model Development

The relationship between VIs and foliar N concentration was assessed through Pearson correlations (r) and a test of significance at the level of *p* ≤ 0.05 in SPSS 20.0. When a significant correlation was present between VIs and foliar N concentration, a linear model was tested for regression analysis. To study the linear relationship between variables, the partial least squares regression (PLSR) model was constructed, and the data were computed using Python 3.7.3. The standard PLSR equation can be expressed as follows:


(1) $$y=\beta_{1}\,x_{1}+\beta_{2}\,x_{2}+\ldots +\beta_{i}\,x_{i}+\varepsilon$$

where *y* = response variable that represents foliar N,

x_*i*_ = predictor variable representing spectral data,

β_*i*_ = the estimated weighted regression coefficient, and

ε = error vector.

Partial least squares regression is the most popular linear model that has applications in a wide range of fields (Kamruzzaman et al., 2012; Zhang et al., 2015; Li et al., 2016, 2018). It is a robust and powerful modeling procedure in comparison to many traditional multivariate regression models (Sun et al., 2017; Li et al., 2018). It can efficiently analyze data including several multi-collinear variables. In the present study, we used N concentration as the dependent variable and VI as an independent variable. The model was performed by the leave-one-out cross-validation procedure to assess the validation of model quality. In this method, all samples excluding one were employed to build a validation model, which was subsequently utilized to predict the rest of the samples. The cross-validation method evaluates the predictive capability of a model and is statistically a more comprehensive method for selecting an appropriate number of components to be retained in the model. The performance of PLSR was measured with the help of the *R*^2^ (coefficient of determination) and the RMSE (root-mean-square error), which is calculated as follows:


(2) $$RMSE\left(\%\right)=\sqrt{\frac{\sum_{i=1}^{n}\left(Y\,i-y\,i\right)}{n}}$$

where Yi = N concentration of the ith sample calculated by the equation,

yi = N concentration analyzed in the lab for “i” sample, and

*n* = total number of samples.

**FIGURE 2:**
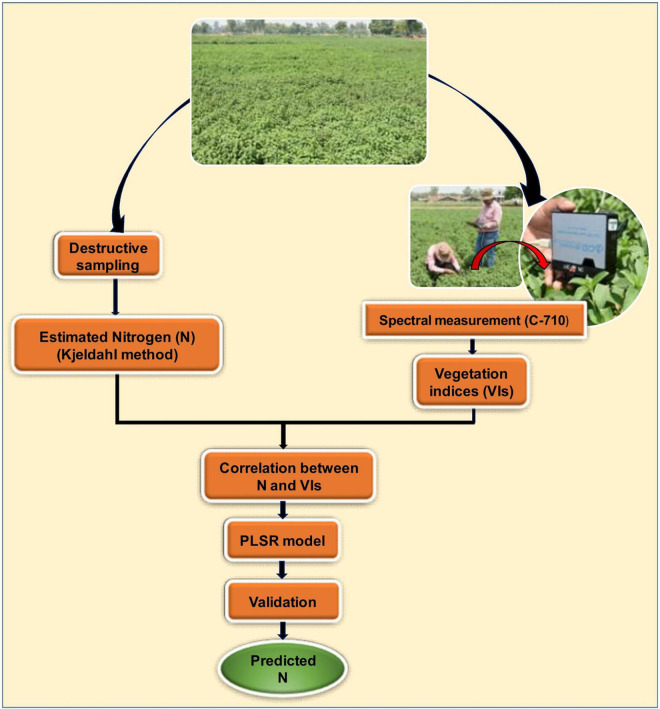
Schematic representation of the methodology adopted in this study.

The precision of the model was considered being more accurate when the *R*^2^ value was close to 1 and the RMSE value was close to 0 (Zheng et al., 2018). A detailed methodology adopted in this study for estimating foliar N concentration is shown in Figure 2.

## Results

The performance of different VI (NDRE, reNDVI, SR, GRVI, CCCI, PRI, CI_*Green*_, CI_*Red Edge*_, CCI, NPCI, and SIPI) derived from spectral reflectance studies was evaluated for estimating foliar N concentration. It was observed that all the VIs significantly and positively correlated with foliar N concentration (Table 2). In other words, these VIs were good indicators of N concentration in menthol mint plants. Based on the magnitude of Pearson correlation, we categorized the estimates as high (<0.80), medium (0.70–0.80), and low correlation (0.60–0.70) estimates. The highest correlation between VIs and N concentrations was retrieved by NDRE (0.89), followed by reNDVI (0.84) and SR (0.83). Medium correlation with N concentration was shown by the VIs GRVI (0.78), CCCI (0.76), PRI (0.71), CI_*Green*_ (0.71), and CI_*Red Edge*_ (0.71), while a weak correlation was demonstrated by CCI (0.68), NPCI (0.68), and SIPI (0.64).

**TABLE 2:** Pearson correlation coefficient (r) between vegetation indices (VIs) and foliar nitrogen concentrations (N).

	NDRE	reNDVI	SR	GRVI	CCCI	CI_Green_	PRI	CI_Red Edge_	CCI	NPCI	SIPI
N (mg 100 mgDW^–1^)	0.89*	0.84*	0.83*	0.78*	0.76*	0.71*	0.71*	0.71*	0.68*	0.68*	0.64*

*Significant correlations at 0.05 level. NDRE, normalized difference red edge; reNDVI, red edge normalized difference vegetation index; SR, simple ratio; GRVI, green–red vegetation index; CCCI, canopy chlorophyll content index; CI_Green_, green chlorophyll index; PRI, photochemical reflectance index; CI_Red Edge_, red edge chlorophyll index; CCI, canopy chlorophyll index; NPCI, normalized pigment chlorophyll ratio index; SIPI, structure insensitive pigment index.

**FIGURE 3:**
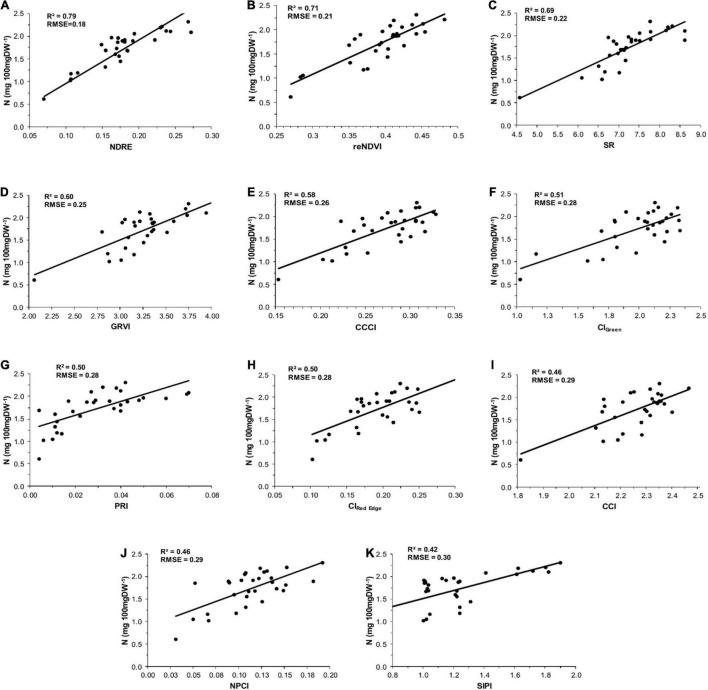
Relationship between foliar nitrogen concentrations and vegetation indexes: **(A)** normalized difference red edge (NDRE), **(B)** red edge normalized difference vegetation index (reNDVI), **(C)** simple ratio (SR), **(D)** green-red vegetation index (GRVI), **(E)** canopy chlorophyll content index (CCCI), **(F)** green chlorophyll index (CI_*Green*_), **(G)** photochemical reflectance index (PRI), **(H)** red edge chlorophyll index (CI_*Red Edge*_), **(I)** canopy chlorophyll index (CCI), **(J)** normalized pigment chlorophyll ratio index (NPCI), and **(K)** structure insensitive pigment index (SIPI) in menthol mint.

According to Zheng et al. (2018), lower RMSE and higher *R*^2^ values obtained from PLSR analysis indicate better estimation efficiency of the foliar N content. In this context, NDRE index showed a very strong correlation with foliar N concentration (*R*^2^ = 0.79, RMSE = 0.18) and provides the most accurate results among all the VIs used in this study (Figure 3). In comparison, reNDVI (*R*^2^ = 0.71, RMSE = 0.21), SR (*R*^2^ = 0.69, RMSE = 0.22), GRVI (*R*^2^ = 0.60, RMSE = 0.25), CCCI (*R*^2^ = 0.58, RMSE = 0.26), CI_*Green*_ (*R*^2^ = 0.51, RMSE = 0.28), PRI (*R*^2^ = 0.50, RMSE = 0.28), and CI_*Red Edge*_ (*R*^2^ = 0.50, RMSE = 0.28) also provided good estimations for N concentration. Nevertheless, a weak correlation was observed between foliar N and VIs SIPI (*R*^2^ = 0.42, RMSE = 0.30), NPCI (*R*^2^ = 0.46, RMSE = 0.29), and CCI (*R*^2^ = 0.46, RMSE = 0.29).

**FIGURE 4:**
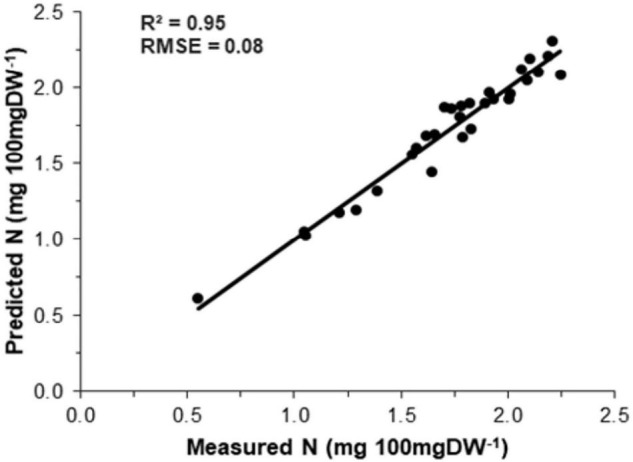
Relationship between measured and predicted nitrogen concentrations in menthol mint.

**FIGURE 5:**
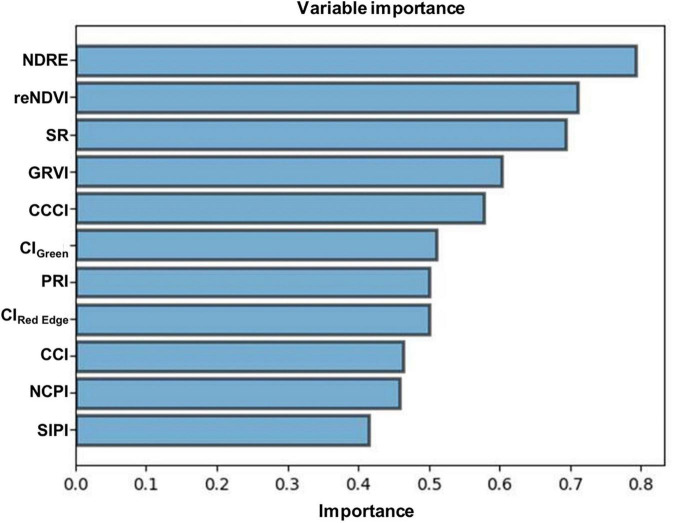
Independent variables with their respective importance values.

The association between measured N (Kjeldahl method) and predicted N (model) is shown in Figure 4. The precision and accuracy of the model were found to be very high (*R*^2^ = 0.95, RMSE = 0.08), suggesting that predicted N concentration is strongly correlated with measured N concentration. The sensitivity study was also performed using spectral variables to calculate their specific importance toward the reliability of the PLSR model. The outcomes (Figure 5) showed that all the independent variables significantly affected the model output.

## Discussion

Fertilizers play a vital role in improving crop yield and productivity. Among them, nitrogenous fertilizers are important to mineral nutrition and significantly affect the biological process of the plant, right from its germination stage till maturity, and thus have a considerable impact on crop yields. Excess amount of N fertilizers not only increases the cost of cultivation, but is also harmful to soil, environment, and human health (Ahmed et al., 2017; Rhezali and Lahlali, 2017). Hence, the accurate estimation of the N requirements of the crop is crucial for optimizing N fertilizer management. Previous studies have shown that N content is strongly correlated with plant photosynthetic activities (Baret et al., 2007; Maathuis, 2009; Schlemmer et al., 2013; Yao et al., 2015). Over the past years, the chlorophyll content of leaves was frequently used as an index for detecting nutrient and N status in plants (Dey et al., 2016). Keeping in view, the key objective of this investigation was to explore a rapid and non-invasive method for estimating foliar N concentration in menthol mint plants. In the present study, destructive foliar N concentration was measured through the Kjeldahl method and spectral reflectance by Miniature Leaf Spectrometer C-710 (CID Bio-Science). Using reflectance data, NDRE, reNDVI, SR, GRVI, CCCI, PRI, CI_*Green*_, CI_*Red Edge*_, CCI, NPCI, and SIPI were measured and further used to determine the foliar N concentration. The Pearson correlation analysis showed that all the VIs were significantly and positively correlated to N concentration, signifying that these VIs were good indicators of N concentration in menthol mint plants. The highest correlation (r) between VIs and N concentrations was retrieved by NDRE, followed by reNDVI and SR. The VIs GRVI, CCCI, PRI, CI_*Green*_, and CI_*Red Edge*_ showed a medium correlation, while VIs CCI, NPCI, and SIPI exhibited a low correlation with N concentration. Similar findings were also reported by previous studies (Oliveira et al., 2017; Shaver et al., 2017).

Following the presence of a significant correlation, the PLSR model was used to evaluate the linear relationship between variables, which revealed that the NDRE vegetation index has a significant correlation with foliar N concentration and provides the most precise results among all the VIs used in this analysis. This is in agreement with the findings of the previous studies (Hansen and Schjoerring, 2003; Li et al., 2014, 2018; Oliveira et al., 2017; Perry et al., 2018; Singh et al., 2019). Studies have shown that NDRE is a better indicator of crop health and is generally found to be sensitive toward the crops having high levels of chlorophyll and nitrogen content (Gitelson et al., 1996; Eitel et al., 2008; Cammarano et al., 2014; Liu et al., 2016). The linear relationship between VIs and foliar N also showed that the VIs based on the reflectance of the red-edge band (i.e., NDRE and reNDVI) had higher *R*^2^ values and lower RMSE values, which indicated that VIs based on the reflectance of the red-edge band were more sensitive toward the estimation of foliar N status than the other VIs evaluated in this study. These results are in agreement with the findings of Yu et al. (2013), who found that the red-edge-based VIs were more sensitive to plant N concentration. The reason behind this is some degree of saturation may be appeared due to only red-edge band. Similarly, we also found a strong correlation between NDRE, GRVI, and SR indices and nitrogen content in the menthol mint crops in our preliminary study (Singh et al., 2019). However, a weak correlation between foliar N and VIs was observed for SIPI, NPCI, and CCI. Wu et al. (2008) suggested that SIPI and NPCI appear to quickly saturate at low chlorophyll levels and become insensitive to high chlorophyll content, which might be the reason for a weak correlation between SIPI and NPCI indices and foliar N concentration. Chlorophyll is not only used as an alternate indicator for the estimation of leaf nitrogen content, but is also an indispensable indicator of N deficiency in plants (Cerovic et al., 2012). Moreover, in the present study, the performance of chlorophyll/carotenoid-based index (CCI) in estimating the foliar N concentration was found to be poor among all the VIs; nevertheless, a strong linear relationship between CCI and N concentration was reported by several researchers (Peng et al., 1993; Van den Berg and Perkins, 2004; Pal et al., 2012; Mace and Mills, 2015).

Analyzing the model’s precision is an essential part of developing regression models because it describes how well the model performs in its predictions. In this study, the model precision and accuracy between measured N and predicted N concentration was found to be very high, indicating that the predicted N is strongly correlated with the measured N concentration. The results of the sensitivity analysis of the PLSR model revealed that all the independent variables had a significant impact on the model output.

## Conclusion

The revolutionary scientific and technological development, particularly in the field of remote sensing, has greatly influenced agricultural practices in the 21st century. Remote sensing techniques have been proved to be a promising method for monitoring health, nutrient evaluation, and yield prediction of crops. Constant monitoring of plant N content might be useful for farmers to expand their farming practices and facilitate real-time observation for site-specific fertilizer management, which would favor novel inventions in the coming years. Hence, the application of excess fertilizers should be reduced to prevent destructive consequences on the environment, which can simultaneously provide supportable benefits to the production capital. It is apparent from this study that NDRE and reNDVI are the most sensitive VIs for the estimation of foliar N concentration in menthol mint plants. This finding suggests that N deficiencies encountered during the growth of the menthol mint crops can be detected by calculating NDRE and reNDVI vegetative indices. In comparison, SR, GRVI, CCCI, CI_*Green*_, PRI, and CI_*Red Edge*_ indices also provided good estimations, whereas SIPI, NPCI, and CCI indices showed a weak correlation with foliar N concentration. The results of the sensitivity analysis of the PLSR model revealed that all the independent variables had a significant impact on the model output. The model precision and accuracy between measured N and predicted N concentration was found to be very high, indicating that the predicted N is strongly correlated with the measured N concentration. We firmly believe that this information can be used as an indicator for recommending the application of precise amounts of nitrogenous fertilizers.

## Data Availability Statement

The raw data supporting the conclusions of this article will be made available by the authors, without undue reservation.

## Author Contributions

MS conceived and designed the experiment, and corrected and finalized the manuscript. PP, SS, and MSK conducted the experiments and collected the data. PP analyzed the data and wrote the manuscript. SS helped in manuscript preparation. All authors reviewed and agreed on the final version.

## Conflict of Interest

The authors declare that the research was conducted in the absence of any commercial or financial relationships that could be construed as a potential conflict of interest. The reviewer RL declared a shared affiliation with the authors to the handling editor at the time of review.

## Publisher’s Note

All claims expressed in this article are solely those of the authors and do not necessarily represent those of their affiliated organizations, or those of the publisher, the editors and the reviewers. Any product that may be evaluated in this article, or claim that may be made by its manufacturer, is not guaranteed or endorsed by the publisher.

## Abbreviations

NDRI: normalized difference red edge
reNDVI: red edge normalized difference vegetation index
SR: simple ratio
GRVI: green–red vegetation index
CCCI: canopy chlorophyll content index
PRI: photochemical reflectance index
CI_*Green*_: green chlorophyll index
CI_*Red Edge*_: red edge chlorophyll index
CCI: canopy chlorophyll index
NPCI: normalized pigment chlorophyll ratio index
SIPI: structure insensitive pigment index
PLSR: partial least squares regression
VIs: vegetation indices
NIR: near-infrared
SWIR: shortwave-infrared.

## References

[B1] Ahmed M. Rauf M. Mukhtar Z. Saeed N. A. (2017). Excessive use of nitrogenous fertilizers: an unawareness causing serious threats to environment and human health. *Environ. Sci. Pollut. Res.* 24 26983–26987. 10.1007/s11356-017-0589-7 29139074

[B2] Baret F. Houlès V. Guérif M. (2007). Quantification of plant stress using remote sensing observations and crop models: the case of nitrogen management. *J. Exp. Bot.* 58 869–880. 10.1093/jxb/erl231 17220515

[B3] Birth G. S. McVey G. R. (1968). Measuring the color of growing turf with a reflectance spectro-radiometer. *Agron. J.* 60 640–643. 10.2134/agronj1968.00021962006000060016x

[B4] Biswas D. K. Ma B. L. (2016). Effect of nitrogen rate and fertilizer nitrogen source on physiology, yield, grain quality, and nitrogen use efficiency in corn. *Can. J. Plant Sci.* 96 392–403. 10.1139/cjps-2015-0186

[B5] Cammarano D. Fitzgerald G. J. Casa R. Basso B. (2014). Assessing the robustness of vegetation indices to estimate wheat N in Mediterranean environments. *Remote Sens.* 6 2827–2844. 10.3390/rs6042827

[B6] Cerovic Z. G. Masdoumier G. Ghozlen N. B. Latouche G. (2012). A new optical leaf-clip meter for simultaneous non-destructive assessment of leaf chlorophyll and epidermal flavonoids. *Physiol. Plant* 146 251–260. 10.1111/j.1399-3054.2012.01639.x 22568678 PMC3666089

[B7] Chen P. Haboudane D. Tremblay N. Wang J. Vigneault P. Li B. (2010). New spectral indicator assessing the efficiency of crop nitrogen treatment in corn and wheat. *Remote Sens. Environ.* 114 1987–1997. 10.1016/j.rse.2010.04.006

[B8] Clevers J. G. Gitelson A. A. (2013). Remote estimation of crop and grass chlorophyll and nitrogen content using red-edge bands on Sentinel-2 and-3. *Int. J. Appl. Earth Obs. Geoinform.* 23 344–351. 10.1016/j.jag.2012.10.008

[B9] Dey A. K. Sharma M. Meshram M. R. (2016). An analysis of leaf chlorophyll measurement method using chlorophyll meter and image processing technique. *Procedia Comput. Sci.* 85 286–292. 10.1186/s13007-015-0048-8 25793008 PMC4365514

[B10] Dobermann A. Cruz P. S. Cassman K. G. (1996). Fertilizer inputs, nutrient balance, and soil nutrient-supplying power in intensive, irrigated rice systems. I. Potassium uptake and K balance. *Nutr. Cycling Agroecosyst.* 46 1–10. 10.1007/bf00210219

[B11] Du L. Gong W. Shi S. Yang J. Sun J. Zhu B. (2016). Estimation of rice leaf nitrogen contents based on hyperspectral LIDAR. *Int. J. Appl. Earth Obs. Geoinform.* 44 136–143. 10.1364/OE.25.006539 28381001

[B12] Eitel J. U. H. Long D. S. Gessler P. E. Hunt E. R. (2008). Combined spectral index to improve ground-based estimates of nitrogen status in dryland wheat. *Agron. J.* 100 1694–1702. 10.2134/agronj2007.0362

[B13] Feng W. Guo B. B. Wang Z. J. He L. Song X. Wang Y. H. (2014). Measuring leaf nitrogen concentration in winter wheat using double-peak spectral reflection remote sensing data. *Field Crops Res.* 159 43–52. 10.1016/j.fcr.2014.01.010

[B14] Fitzgerald G. J. Rodriguez D. Christensen L. K. Belford R. Sadras V. O. Clarke T. R. (2006). Spectral and thermal sensing for nitrogen and water status in rainfed and irrigated wheat environments. *Precis. Agric.* 7 233–248. 10.1007/s11119-006-9011-z

[B15] Gamon J. Serrano L. Surfus J. S. (1997). The photochemical reflectance index: an optical indicator of photosynthetic radiation use efficiency across species, functional types, and nutrient levels. *Oecologia* 112 492–501. 10.1007/s004420050337 28307626

[B16] Gitelson A. A. Gritz Y. Merzlyak M. N. (2003). Relationships between leaf chlorophyll content and spectral reflectance and algorithms for non-destructive chlorophyll assessment in higher plant leaves. *J. Plant Physiol.* 160 271–282. 10.1078/0176-1617-00887 12749084

[B17] Gitelson A. A. Kaufman Y. J. Merzlyak M. N. (1996). Use of a green channel in remote sensing of global vegetation from EOS-MODIS. *Remote Sens. Environ.* 58 289–298. 10.1016/s0034-4257(96)00072-7

[B18] Gitelson A. A. Merzlyak M. N. (1994). Quantitative estimation of chlorophyll-ausing reflectance spectra: experiments with autumn chestnut and maple leaves. *J. Photochem. Photobiol. B* 22 247–252. 10.1016/1011-1344(93)06963-4

[B19] Gitelson A. A. Vina A. Ciganda V. Rundquist D. C. Arkebauer T. J. (2005). Remote estimation of canopy chlorophyll content in crops. *Geophys. Res. Lett.* 32 93–114.

[B20] Hansen P. M. Schjoerring J. K. (2003). Reflectance measurement of canopy biomass and nitrogen status in wheat crops using normalized difference vegetation indices and partial least squares regression. *Remote Sens. Environ.* 86 542–553. 10.1016/s0034-4257(03)00131-7

[B21] Herrmann I. Karnieli A. Bonfil D. J. Cohen Y. Alchanatis V. (2010). SWIR-based spectral indices for assessing nitrogen content in potato fields. *Int. J. Remote Sens.* 31 5127–5143. 10.1080/01431160903283892

[B22] Jay S. Maupas F. Bendoula R. Gorretta N. (2017). Retrieving LAI, chlorophyll and nitrogen contents in sugar beet crops from multi-angular optical remote sensing: comparison of vegetation indices and PROSAIL inversion for field phenotyping. *Field Crops Res.* 210 33–46. 10.1016/j.fcr.2017.05.005

[B23] Ju X. T. Kou C. L. Zhang F. S. Christie P. (2006). Nitrogen balance and groundwater nitrate contamination: comparison among three intensive cropping systems on the North China Plain. *Environ. Pollut.* 143 117–125. 10.1016/j.envpol.2005.11.005 16364521

[B24] Kamruzzaman M. ElMasry G. Sun D. W. Allen P. (2012). Prediction of some quality attributes of lamb meat using near-infrared hyperspectral imaging and multivariate analysis. *Anal. Chim. Acta* 714 57–67. 10.1016/j.aca.2011.11.037 22244137

[B25] Kattge J. (2002). *Zur Bedeutung von Stickstoff für den CO2—Düngeeffekt.* Gießen: Justus-Liebig-Universität, 285.

[B26] Khan M. S. Semwal M. Sharma A. Verma R. K. (2020). An artificial neural network model for estimating *Mentha* crop biomass yield using Landsat 8 OLI. *Precis. Agric.* 21 18–33. 10.1007/s11119-019-09655-9

[B27] Lang C. A. (1958). Simple microdetermination of Kjeldahl nitrogen in biological materials. *Anal. Chem.* 30 1692–1694. 10.1021/ac60142a038

[B28] Li F. Mistele B. Hu Y. Chen X. Schmidhalter U. (2014). Reflectance estimation of canopy nitrogen content in winter wheat using optimised hyperspectral spectral indices and partial least squares regression. *Eur. J. Agron.* 52 198–209. 10.1016/j.eja.2013.09.006

[B29] Li L. Jakli B. Lu P. Ren T. Ming J. Liu S. (2018). Assessing leaf nitrogen concentration of winter oilseed rape with canopy hyperspectral technique considering a non-uniform vertical nitrogen distribution. *Ind. Crops Prod.* 116 1–14. 10.1016/j.indcrop.2018.02.051

[B30] Li L. Lu J. Wang S. Ma Y. Wei Q. Li X. (2016). Methods for estimating leaf nitrogen concentration of winter oilseed rape (*Brassica napus* L.) using in situ leaf spectroscopy. *Ind. Crops Prod.* 91 194–204. 10.1016/j.indcrop.2016.07.008

[B31] Liu Y. Cheng T. Zhu Y. Tian Y. Cao W. Yao X. (2016). “Comparative analysis of vegetation indices, non-parametric and physical retrieval methods for monitoring nitrogen in wheat using UAV-based multispectral imagery,” in *2016 IEEE Int. Geosci. Remote Sens. Symp. (IGARSS)*, Beijing: IEEE, 7362–7365.

[B32] Maathuis F. J. (2009). Physiological functions of mineral macronutrients. *Curr. Opin. Plant Biol.* 12 250–258. 10.1016/j.pbi.2009.04.003 19473870

[B33] Mace K. C. Mills N. J. (2015). Response of walnut aphid populations to increasing foliar nitrogen content. *Agric. For. Entomol.* 17 277–284. 10.1111/afe.12103

[B34] Main R. Cho M. A. Mathieu R. O’Kennedy M. M. Ramoelo A. Koch S. (2011). An investigation into robust spectral indices for leaf chlorophyll estimation. *ISPRS J. Photogramm. Remote Sens.* 66 751–761. 10.1016/j.isprsjprs.2011.08.001

[B35] Oliveira L. F. R. D. Oliveira M. L. R. D. Gomes F. S. Santana R. C. (2017). Estimating foliar nitrogen in *Eucalyptus* using vegetation indexes. *Sci. Agric.* 74 142–147. 10.1590/1678-992x-2015-0477

[B36] Pal P. K. Singh R. D. Prasad R. (2012). Non-destructive estimation of chlorophyll and nitrogen content in leaf of *Rosa damascena* (Mill). *Soil Sci. Plant Nutr.* 58 604–610. 10.1080/00380768.2012.723993

[B37] Peng S. Garcia F. V. Laza R. C. Cassman K. G. (1993). Adjustment for specific leaf weight improves chlorophyll meter’s estimate of rice leaf nitrogen concentration. *Agron J.* 85 987–990. 10.2134/agronj1993.00021962008500050005x

[B38] Peñuelas J. Baret F. Filella I. (1995). Semi-empirical indices to assess carotenoids/chlorophyll a ratio from leaf spectral reflectance. *Photosynthetica* 31 221–230.

[B39] Peñuelas J. Gamon J. A. Fredeen A. L. Merino J. Field C. B. (1994). Reflectance indices associated with physiological changes in nitrogen- and water-limited sunflower leaves. *Remote Sens. Environ.* 48 135–146.

[B40] Perry E. M. Goodwin I. Cornwall D. (2018). Remote sensing using canopy and leaf reflectance for estimating nitrogen status in red-blush pears. *Hort. Sci.* 53 78–83. 10.21273/hortsci12391-17

[B41] Rhezali A. Lahlali R. (2017). Nitrogen (N) mineral nutrition and imaging sensors for determining N status and requirements of maize. *J. Imaging* 3 51–60. 10.3390/jimaging3040051

[B42] Rouse J. W. Haas R. H. Schell J. A. Deering D. W. (1973). “Monitoring vegetation systems in the great plains with ERTS. 3rd ERTS symposium,” in *NASA SP- 351*, Washington DC, 309–317.

[B43] Schlemmer M. Gitelson A. Schepers J. Ferguson R. Peng Y. Shanahan J. (2013). Remote estimation of nitrogen and chlorophyll contents in maize at leaf and canopy levels. *Int. J. Appl. Earth Obs. Geoinform.* 25 47–54. 10.1016/j.jag.2013.04.003

[B44] Serrano L. Penuelas J. Ustin S. L. (2002). Remote sensing of nitrogen and lignin in Mediterranean vegetation from AVIRIS data: Decomposing biochemical from structural signals. *Remote Sens. Environ.* 81 355–364. 10.1016/s0034-4257(02)00011-1

[B45] Shaver T. M. Kruger G. R. Rudnick D. R. (2017). Crop canopy sensor orientation for late season nitrogen determination in corn. *J. Plant Nutr.* 40 2217–2223. 10.1080/01904167.2017.1346681

[B46] Sims D. A. Luo H. Y. Hastings S. Oechel W. C. Rahman A. F. Gamon J. A. (2006). Parallel adjustments in vegetation greenness and ecosystem CO2 exchange in response to drought in a southern California chaparral ecosystem. *Remote Sens. Environ.* 103 289–303. 10.1016/j.rse.2005.01.020

[B47] Singh S. Khan M. S. Pandey P. Semwal M. (2019). “Estimating foliar nitrogen using hyperspectral Vegetation indices in Mentha,” in *National Conference ‘Innovations in Geospatial Technology for Sustainable Development With Special Emphasis on NER*, Shillong, 265–266.

[B48] Song Y. Wang J. (2016). “Soybean canopy nitrogen monitoring and prediction using ground based multispectral remote sensors,” in *2016 IEEE Int. Geosci. Remote Sens. Symp. (IGARSS)*, Beijing: IEEE, 6389–6392.

[B49] Sun J. Yang J. Shi S. Chen B. Du L. Gong W. (2017). Estimating rice leaf nitrogen concentration: influence of regression algorithms based on passive and active leaf reflectance. *Remote Sens.* 9:951. 10.3390/rs9090951

[B50] Tarpley L. Reddy K. R. Sassenrath-Cole G. F. (2000). Reflectance indices with precision and accuracy in predicting cotton leaf nitrogen concentration. *Crop Sci.* 40 1814–1819. 10.2135/cropsci2000.4061814x

[B51] Tian Y. C. Yao X. Yang J. Cao W. X. Hannaway D. B. Zhu Y. (2011). Assessing newly developed and published vegetation indices for estimating rice leaf nitrogen concentration with ground-and space-based hyperspectral reflectance. *Field Crops Res.* 120 299–310. 10.1016/j.fcr.2010.11.002

[B52] Tucker C. J. (1979). Red and photographic infrared linear combinations for monitoring vegetation. *Remote Sens. Environ.* 8 127–150. 10.1016/0034-4257(79)90013-0

[B53] Van den Berg A. K. Perkins T. D. (2004). Evaluation of portable chlorophyll meter to estimate chlorophyll and nitrogen contents in sugar maple (*Acer saccharum* Marsh.) leaves. *For. Ecol. Manage.* 200 113–117. 10.1016/j.foreco.2004.06.005

[B54] Wu C. Niu Z. Tang Q. Huang W. (2008). Estimating chlorophyll content from hyperspectral vegetation indices: modeling and validation. *Agric. For. Meteorol.* 148 1230–1241. 10.1016/j.agrformet.2008.03.005

[B55] Yang J. Du L. Sun J. Zhang Z. Chen B. Shi S. (2016). Estimating the leaf nitrogen content of paddy rice by using the combined reflectance and laser-induced fluorescence spectra. *Opt. Express* 24 19354–19365. 10.1364/OE.24.019354 27557214

[B56] Yao X. Huang Y. Shang G. Zhou C. Cheng T. Tian Y. (2015). Evaluation of six algorithms to monitor wheat leaf nitrogen concentration. *Remote Sens.* 7 14939–14966. 10.3390/rs71114939

[B57] Yu K. Li F. Gnyp M. L. Miao Y. Bareth G. Chen X. (2013). Remotely detecting canopy nitrogen concentration and uptake of paddy rice in the Northeast China Plain. *ISPRS J. Photogramm. Remote Sens.* 78 102–115. 10.1016/j.isprsjprs.2013.01.008

[B58] Zhang C. Liu F. Kong W. He Y. (2015). Application of visible and near-infrared hyperspectral imaging to determine soluble protein content in oilseed rape leaves. *Sensors* 15 16576–16588. 10.3390/s150716576 26184198 PMC4541894

[B59] Zheng H. Cheng T. Li D. Zhou X. Yao X. Tian Y. (2018). Evaluation of RGB, color-infrared and multispectral images acquired from unmanned aerial systems for the estimation of nitrogen accumulation in rice. *Remote Sens.* 10:824. 10.3390/rs10060824

[B60] Zhou K. Cheng T. Zhu Y. Cao W. Ustin S. L. Zheng H. (2018). Assessing the impact of spatial resolution on the estimation of leaf nitrogen concentration over the full season of paddy rice using near-surface imaging spectroscopy data. *Front. Plant Sci.* 9:964. 10.3389/fpls.2018.00964 30026750 PMC6041568

[B61] Zhu Y. Tian Y. Yao X. Liu X. Cao W. (2007). Analysis of common canopy reflectance spectra for indicating leaf nitrogen concentrations in wheat and rice. *Plant Prod. Sci.* 10 400–411. 10.1626/pps.10.400

